# Association between anemia and diabetic lower extremity ulcers among US outpatients in the National Health and Nutrition Examination Survey: a retrospective cross-sectional study

**DOI:** 10.3389/fendo.2024.1387218

**Published:** 2024-08-29

**Authors:** Jinmin Cao, Jingpei Wang, Saiqian Zhang, Guiyun Gao

**Affiliations:** Department of Dermatology, Hunan Aerospace Hospital, Changsha, China

**Keywords:** hemoglobin, anemia, diabetic lower extremity ulcers, NHANES, cross-sectional study

## Abstract

**Purpose:**

The aim of this study was to explore the relationship between hemoglobin levels, anemia and diabetic lower extremity ulcers in adult outpatient clinics in the United States.

**Methods:**

A retrospective cross-sectional study was conducted on 1673 participants in the National Health and Nutrition Examination Survey (NHANES) from 1999 to 2004. Three logistic regression models were developed to evaluate the relationship between anemia and diabetic lower extremity ulcers. Model 1 adjusted for demographic and socioeconomic variables (age, sex, race and ethnicity, educational level, family income, and marital status). Model 2 included additional health-related factors (BMI, cardiovascular disease, stroke, family history of diabetes, hyperlipidemia, alcohol and smoking status). Model 3 further included clinical and laboratory variables (HbA1c, CRP, total cholesterol, and serum ferritin levels). Stratified analyses were also conducted based on age, sex, HbA1c level, body mass index (BMI), and serum ferritin level.

**Results:**

The study included 1673 adults aged 40 years and older, with a mean age of 64.7 ± 11.8 years, of whom 52.6% were male. The prevalence of diabetic lower extremity ulcers (DLEU) was 8.0% (136 participants). Anemia was found in 239 participants, accounting for 14% of the study group. Model 1 showed an OR of 2.02 (95% CI=1.28~3.19) for anemia, while Model 2 showed an OR of 1.8 (95% CI=1.13~2.87). In Model 3, the OR for DFU in patients with anemia was 1.79 (95% CI=1.11~2.87). Furthermore, when serum ferritin was converted to a categorical variable, there was evidence of an interaction between DLEU status and serum ferritin in increasing the prevalence of DLEU.

**Conclusion:**

After adjusting for confounding variables, higher levels of anemia were proportionally associated with an increased risk of incident DLEU. These results suggest that monitoring T2DM patients during follow-up to prevent the development of DLEU may be important. However, further prospective studies are needed to provide additional evidence.

## Background

The International Diabetes Federation (IDF) has recently published data indicating that there has been a 16% increase (74 million) in the number of adults living with diabetes since 2019. Currently, approximately 537 million adults are affected by this condition. In 2021, T2DM was estimated to cause over 6.7 million deaths in the population aged 20-79 (1). Diabetic foot ulcers are one of the common and serious complications of diabetes mellitus, which can cause severe multi-organ complications leading to high mortality rates and significant health costs (2). Approximately 15% of people with diabetes will eventually develop a diabetic foot ulcers, and 14%-24% of these patients will require amputation due to ulcer-related complications (3).

Previous studies have reported that the prevalence of anemia in patients with DFU is over 50% (4). Common risk factor for foot ulceration include peripheral vascular disease, severity of neuropathy, structural foot deformity, concomitant infection, high plantar pressure, poor glycemic control, duration of diabetes, male gender, and presence of other micro and macrovascular complications. Anemia is also considered a major predictor of the outcome of DFU (5). Research has shown that patients with T2DM are twice as likely to experience anemia compared to those without T2DM (6, 7). The presence of altered microcirculation may exacerbate the negative effects of anemia, hindering ulcer healing and leading to higher rates of amputation and mortality (4, 8–11).

However, there have been no studies conducted on the association between DLEU and anemia in adult outpatients in the United States. The aim of this study was to examine the association between anemia in outpatients with and without DLEU in the NHANES database.

## Materials and methods

### Study population

**Figure 1:**
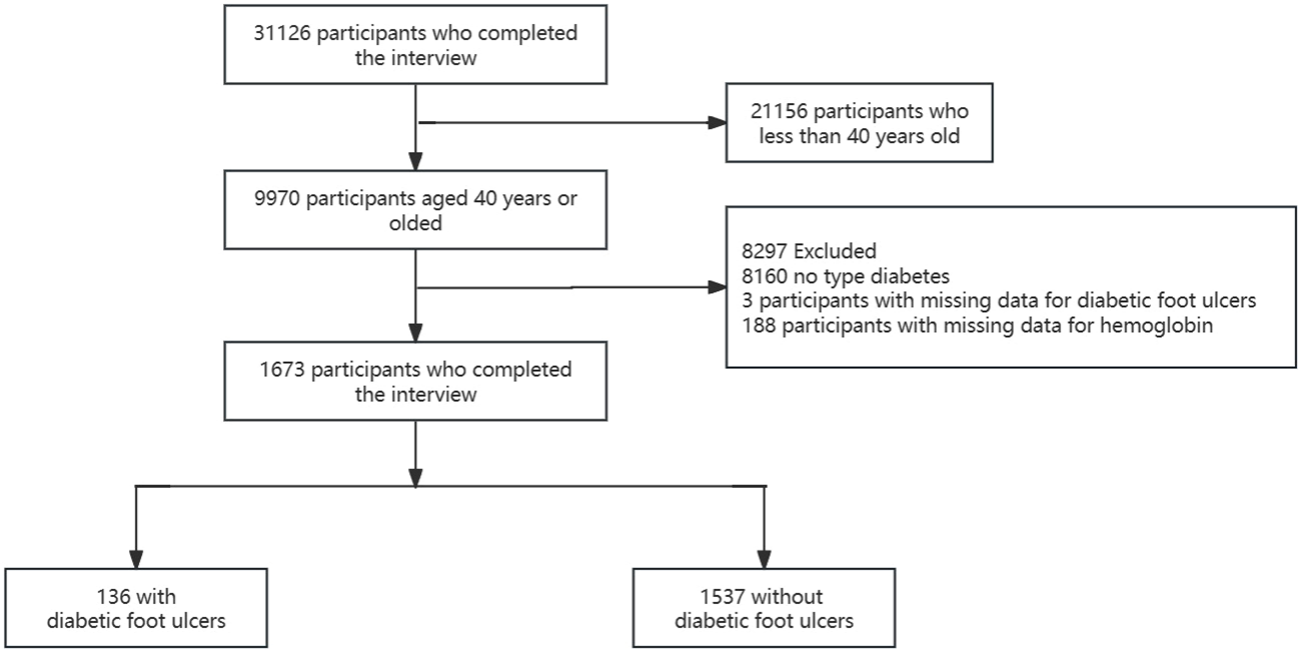
Flowchart of the participant selection. NHANES, National Health and Nutrition Examination Survey.

The National Health and Nutrition Examination Survey (NHANES) was designed to evaluate the health and nutritional status of non-hospitalized Americans using a stratified, multistage approach. The NHANES received approval from the Ethics Review Committee of the National Center for Health Statistics (NCHS), and all participants provided written informed consent prior to participation. This is a retrospective study based on the NHANES database, which contains data on over 31,126 patients from 1999 to 2004. In the study, 9,970 were adults aged 40 years or older who completed the interview and underwent MEC screening. After excluding 8,297 participants who did not have diabetes (n=8160) and those with missing data on diabetes foot ulcers (n=3) and hemoglobin (n=188), the remaining 1,673 participants were included in the analysis (**Figure 1**).

### Ascertainment of diabetic lower extremity t ulcers

The primary outcome variable was the status of diabetic lower extremity ulcers (DLEU), defined by the patient’s self-reported answer to the question in Question Data, ‘Have you had an ulcer or sore on your leg or foot that took more than four weeks to heal?’ Type 2 diabetes mellitus (T2DM) was identified based on the American Diabetes Association criteria and a self-report questionnaire. Participants were considered to have T2DM if they met any of the following criteria (12) (1): Glycated hemoglobin (HbA1c) levels of ≥6.5% (2), Fasting plasma glucose (FPG) levels of ≥126 mg/dL (3), 75 g oral glucose tolerance test (OGTT) levels of ≥11.1mmol/L (4), self-reported physician diagnosis of diabetes, or (5) receipt of oral glucose-lowering medicines or insulin.

### Ascertainment of hemoglobin level, anemia

The NHANES Laboratory/Medical Technologists Procedures Manual (LPM) provides detailed instructions for sample collection and processing. The study employed the Beckman Coulter method for counting and sizing, combined with an automated diluter and mixer for sample processing and a single-beam photometer for hemoglobinometry to derive complete blood count (CBC) parameters. (https://www.cdc.gov/nchs/nhanes/). Anemia was defined by World Health Organization (WHO) as hemoglobin (Hb) levels <13g/dL for males and <12 g/dL for females (7).

### Covariates

Based on the literature, several potential covariates were included in the analysis, such as age, sex, race/ethnicity, education level, marital status, PIR, smoking status, alcohol status, body mass index (BMI), laboratory parameters (total cholesterol and C-reactive protein [CRP], glycosylated hemoglobin [HbA1c], and serum ferritin, and comorbidities (13–16). The comorbidities included family history of diabetes, stroke, coronary heart disease, hyperlipidemia. Marital status was classified as living with a partner, or living alone (15). Family income was divided into three groups according to the poverty income ratio (PIR) as defined by a U.S. government report: low (PIR ≤ 1.3), medium (PIR > 1.3 to 3.5), and high (PIR > 3.5). Alcohol consumption was classified as never (< 12 drinks in lifetime), former (≥12 drinks in 1 year and no drinks in the last year, or no drinks in the previous year but≥12 drinks in lifetime), and current (≥12 drinks and currently drinking). Smoking status was categorized as never (<100 cigarettes in a lifetime), former (≥100 cigarettes but not currently smoking), and current (≥100 cigarettes and currently smoking) (16). Serum ferritin levels were classified as either <100 ng/mL or ≥100 ng/mL, according to previously reported classifications (17). The determination of previous disease (family history of diabetes, stroke, hyperlipidemia, and coronary heart disease) was based on the inquiry in the questionnaire of whether the doctor had been informed of the condition in the past.

### Statistical analysis

The statistical analyses were conducted using R Statistical Software (Version 4.2.2, http://www.R-project.org, The R Foundation) and Free Statistics analysis platform (Version 1.9, Beijing, China, http://www.clinicalscientists.cn/freestatistics). The software is intended for reproducible analysis and interactive computing. A two-sided P value < 0.05 was considered statistically significant.

Normally distributed continuous variables were presented as mean ± SD, and skewed continuous variables were presented as median (interquartile range [IQR]). Categorical variables were expressed as frequencies (%). The Student’s t-test or Mann-Whitney U-test was used to compare continuous variables between groups, depending on the normality of the distribution, and categorical data were compared using the chi-squared or Fisher’s exact test, as appropriate.

Crude model was an unadjusted model. Model 1 was adjusted for age, sex, race and ethnicity, educational level, family income and marital status. Model 2 was developed using model 1 and additional factor such as BMI, coronary heart disease, stroke, family history of diabetes, hyperlipidemia, alcohol and smoking status. Model 3 was then developed using model 2 and additional factor such as HbA1c, CRP, total cholesterol, and serum ferritin. Subgroup analysis was conducted to investigate the correlation between anemia and diabetic lower extremity ulcers based on age, sex, BMI, and HbA1C category (<6.5, ≥6.5) as well as serum ferritin category (<100ng/mL, ≥100ng/mL). The percentage of missing values exceeded 20%. To address this issue, missing data for the covariates were imputed using multiple imputation.

## Results

### Baseline characteristics

**Table 1** displays demographic, socioeconomic, comorbidity, and baseline characteristics by anemia status. The study included 1673 adults aged 40 years and older, with a mean age of 64.7 ± 11.8 years, of whom 52.6% were male. Anemia was found in 239 participants, accounting for 14% of the study group, with a prevalence of 57.3% in women. The prevalence of diabetic lower extremity ulcers was 8.1% (136 participants). The prevalence of diabetic foot ulcers was 12.7% among patients with anemia.

**Table 1:** Characteristics of participants grouped with or without anemia.

	Non-anemia (n=1434)	Anemia (n=239)	p-value
Sex, %			< 0.001
Male	778 (54.3)	102 (42.7)	NA
Female	656 (45.7)	137 (57.3)	NA
Age, years	64.0 ± 11.7	68.8 ± 11.3	< 0.001
Race/ethnicity, %			< 0.001
Non-Hispanic White	420 (29.3)	56 (23.4)	NA
Non-Hispanic Black	68 (4.7)	6 (2.5)	NA
Mexican American	613 (42.7)	68 (28.5)	NA
Other	333 (23.2)	109 (45.6)	NA
Education level, %			0.11
blow high school	694 (48.4)	126 (52.7)	NA
high school	283 (19.7)	53 (22.2)	NA
above high school	457 (31.9)	60 (25.1)	NA
Marital, %			0.002
married or living with partners	886 (61.8)	122 (51)	NA
living alone	548 (38.2)	117 (49)	NA
PIR, %			0.618
Low	500 (34.9)	91 (38.1)	NA
Medium	604 (42.1)	97 (40.6)	NA
High	330 (23)	51 (21.3)	NA
BMI (kg/m2), Mean ± SD	30.9 ± 6.4	31.9 ± 7.5	0.033
coronary heart disease, %			0.003
Yes	157 (10.9)	42 (17.6)	NA
No	1277 (89.1)	197 (82.4)	NA
Stroke, %			0.437
Yes	122 (8.5)	24 (10)	NA
No	1312 (91.5)	215 (90)	NA
Family history of diabetes, %			0.76
Yes	1006 (70.2)	170 (71.1)	NA
No	428 (29.8)	69 (28.9)	NA
Hyperlipidemia, %			0.003
Yes	531 (37)	113 (47.3)	NA
No	903 (63)	126 (52.7)	NA
Alcohol status, %			0.279
Never	284 (19.8)	57 (23.8)	NA
Former	306 (21.3)	53 (22.2)	NA
Now	844 (58.9)	129 (54)	NA
Smoking status, %			0.007
Never	657 (45.8)	118 (49.4)	NA
Former	529 (36.9)	99 (41.4)	NA
Now	248 (17.3)	22 (9.2)	NA
HbA1c%	7.5 ± 1.8	7.1 ± 1.6	0.001
CRP (mg/L), Median (IQR)	0.3(0.2, 0.7)	0.4(0.2, 1.0)	< 0.001
Total cholesterol (mg/dl), mean ± SD	206.8 ± 48.7	191.6 ± 45.2	< 0.001
Ferritin(ng/mL), Median (IQR)	131.5 (66.0, 245.8)	104.0 (52.5, 230.0)	0.004
Diabetic lower extremity ulcers, %			0.007
Yes	106 (7.4)	30 (12.6)	NA
No	1328 (92.6)	209 (87.4)	NA

Mean ± SD for continuous variables: the P-value was calculated by the linear regression model.

Median [IQR] for skewed continuous variables.

% for categorical variables: the P-value was calculated by the chi-square test.

BMI, Body mass index; PIR, Poverty income ratio; HbA1c, Glycosylated hemoglobin; CRP, C-reactive protein.

### Factor associated with diabetic lower extremity ulcers (DLEU)

The univariate ordinal regression analysis results indicated that marital status, BMI, coronary heart disease, family history of diabetes, and hyperlipidemia. (P < 0.1; **Table 2**).

**Table 2:** Univariate Analysis for the Presence of diabetic lower extremity ulcers (DLEU).

Characteristic	OR(95%CI)	P-value
Sex, %		
Male		
Female	0.79 (0.55~1.12)	0.182
Age, years	1 (0.99~1.02)	0.725
Race/ethnicity, %		
Non-Hispanic White		
Non-Hispanic Black	0.91 (0.37~2.23)	0.839
Mexican American	0.94 (0.62~1.43)	0.786
Other	0.78 (0.48~1.26)	0.312
Education level, %		
blow high school		
high school	0.93 (0.58~1.51)	0.779
above high school	1.13 (0.76~1.68)	0.531
Marital, %		
married or living with partners		
living alone	1.38 (0.97~1.97)	0.07
PIR, %		
Low		
Medium	0.69 (0.47~1.03)	0.067
High	0.69 (0.43~1.11)	0.122
BMI (kg/m^2^)	1.04 (1.01~1.06)	0.002
Coronary heart disease; %		
No	1	
Yes	1.67 (1.04~2.66)	0.032
Stroke, %		
No	1	
Yes	1.22 (0.68~2.18)	0.5
Family history of diabetes, %		
No	1	
Yes	1.62 (1.06~2.47)	0.027
Hyperlipidemia, %		
No	1	
Yes	1.37(0.97~1.96)	0.077
Alcohol status, %		
Never	1	
Former	0.84 (0.48~1.47)	0.533
Now	1.04 (0.67~1.63)	0.855
Smoking status, %		
Never	1	
Former	0.97 (0.65~1.44)	0.874
Now	1.3 (0.81~2.09)	0.279
HbA1c,%	1.06 (0.97~1.16)	0.2
CRP	1.05 (0.95~1.15)	0.337
Total cholesterol (mg/dl)	1 (0.99~1)	0.169
Serum Ferritin(ng/mL)	1 (1~1)	0.985
Hemoglobin(g/L)	0.84 (0.75~0.93)	0.001
Anemia		
No	1	
Yes	1.8 (1.17~2.77)	0.008

### Relationship between hemoglobin levels, anemia status and diabetic lower extremity ulcers

**Table 3** presents the odds ratios (OR) and 95% confidence intervals (CI) for the presence of diabetic lower extremity ulcers (DLEU) determined by hemoglobin levels and anemia. When hemoglobin was analyzed as a continuous variable, a significant independent negative association was found between hemoglobin and the risk of DLEU. In the unadjusted model, each 1 unit increase in hemoglobin was associated with a 16% decrease in the presence of DLEU [OR=0.84, 95% CI: (0.75-0.993); p=0.001]. In model 1, 2 and 3, the association between hemoglobin (Hb) and diabetic lower extremity ulcers (DLEU) was marginally significant [OR: 0.74, 95% CI: (0.65-0.84); p<0.001] [OR: 0.76, 95% CI: (0.67-0.86); p<0.001] [OR: 0.76, 95% CI: (0.66-0.86); p<0.001], respectively.

**Table 3:** Relationship between hemoglobin levels, anemia status and diabetic lower extremity ulcers.

		Crude OR(95%CI) p-value	Model 1 OR(95%CI) p-value	Model 2 OR(95%CI) p-value	Model 3 OR(95%CI) p-value
HGB(g/L)		0.84 (0.75~0.93) 0.001	0.74 (0.65~0.84) <0.001	0.76 (0.67~0.86) <0.001	0.76 (0.66~0.86) <0.001
Anemia	No	Reference	Reference	Reference	Reference
	Yes	1.8(1.17~2.77) 0.008	2.02 (1.28~3.19) 0.002	1.8 (1.13~2.87) 0.014	1.79 (1.11~2.87) 0.016

Crude model: Unadjusted model;

Model 1: adjusted for sociodemographic variables (age, sex, race, Marriage, PIR);

Model 2: Model 1 and BMI, Coronary heart disease, stroke, Family history of diabetes, Hyperlipidemia,

Alcohol status, Smoking status;

Model 3: adjusted for Model2, HbA1c, CRP, Total cholesterol, Serum Ferritin, Hemoglobin.

The anemia group had a significantly higher risk of DLEU compared to the non-anemic group [OR: 1.79, 95% CI:(1.11-22.87)]. In **Table 3**, when hemoglobin levels were categorized as anemic versus non-anemia, anemia was found to be positively associated with the risk of diabetic lower extremity ulcers. The odds ratios (OR) for anemia were calculated for Model 1, Model 2, and Model 3, with the crude model as the reference, using multivariable-adjusted regression and 95% confidence intervals (CIs). The odds ratio (OR) for anemia in Model 1 was [OR=2.02,95% CI:(1.28-185 3.19), P=0.002]. In Model 2, the OR for anemia was [OR=1.8,95% CI:(1.13-2.87), P=0.014] and in Model 3, it was [OR=1.79, 95% CI:(1.11-2.87),p=0.016] (**Table 2**). Model 3 exhibited the lowest odds ratio (OR) compared to Model 1, which had the highest OR. This suggests a decreasing trend in the risk of diabetic lower extremity ulcers (DLEU). After conducting multivariate logistic regression analysis and smooth curve fitting, it was found that there is a negative association between hemoglobin levels and DLEU incidence when all potential confounders were taken into account (non-linearity: p=0.572).

### Subgroup analyses of factor influencing the association between anemia and the presence of diabetic lower extremity ulcers

**Figure 2:**
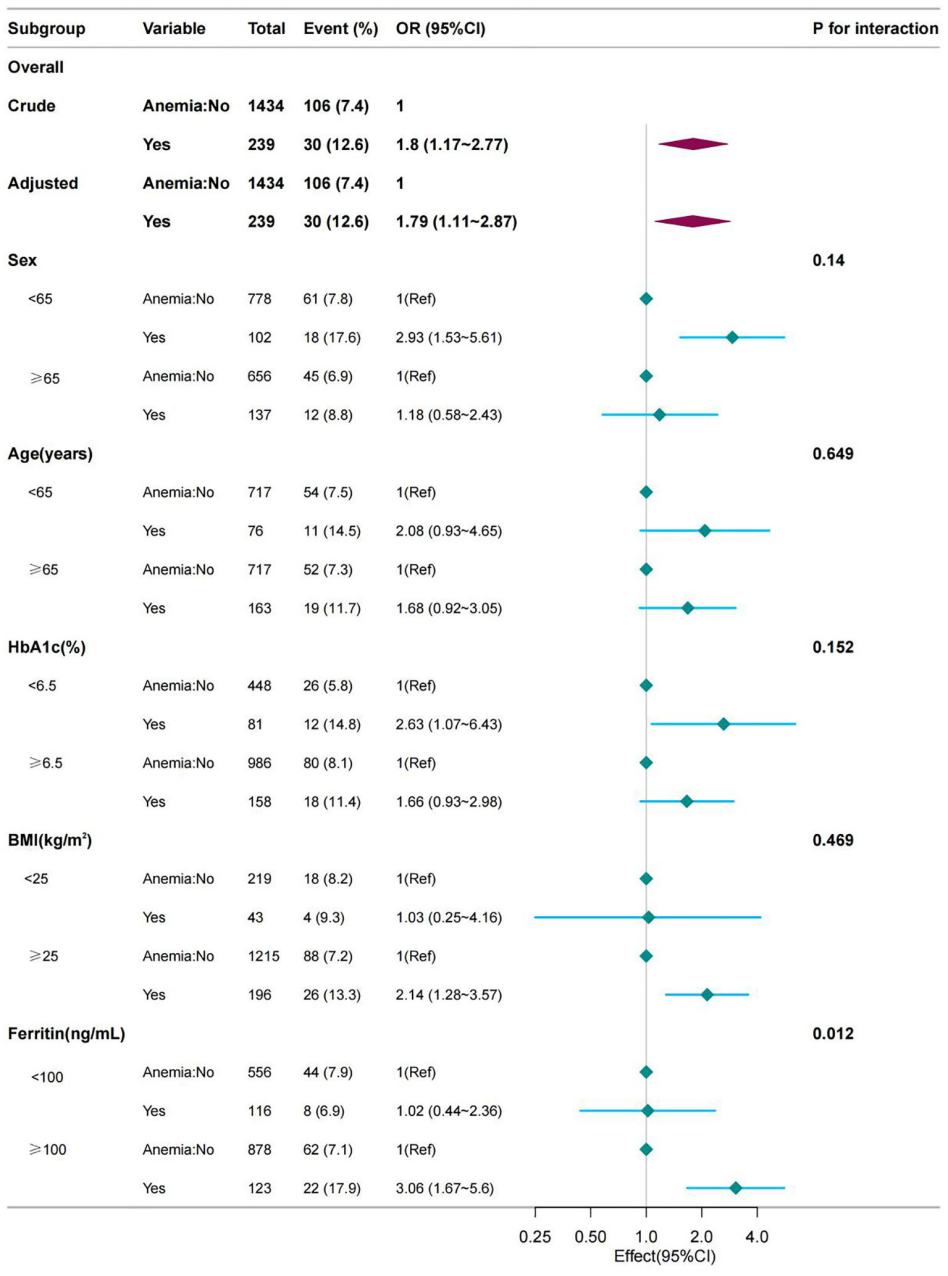
Effect size of anemia on the presence of DLEU in the age, sex, BMI, HbA1c subgroup and serum ferritin level. OR, odds ratio; CI, confidence interval; HGB, hemoglobin.

Stratified analysis was performed in several subgroups to determine the potential effect modifications on the relationship between anemia and DLEU. No significant interactions were found in any subgroup after stratification by sex, age, HbA1c level, and BMI (all P for interaction >0.05). However, results differed between serum ferritin groups for diabetic lower extremity ulcers (P = 0.015 for interaction) (**Figure 2**).

## Discussion

In this cross-sectional study, anemia was found to be positively associated with the incidence of DLEU, and hemoglobin levels were a negative linear association between hemoglobin levels and DLEU Subgroup analysis revealed an interaction between serum ferritin and diabetic lower extremity ulcers, with high serum ferritin identified as a risk factor for diabetic lower extremity ulcers.

In contrast to previous studies that have shown consistency, the incidence of anemia was higher in patients with diabetic foot ulcers than in the non-anemic group (8, 18). Additionally, the prevalence of anemia was higher in women than in men. In this study, the prevalence rate of anemia in the DLEU group was 12.6%, which is higher than the rate in the non-DLEU group (7.4%). DFU can lead to high amputation and mortality rates, particularly in older patients with low hemoglobin levels (10). The more severe the anemia, the greater the impact on ulcer healing, and the higher the amputation rate and mortality (19, 20). Severe anemia can significantly impact ulcer healing and increase the rates of amputation and mortality (8, 21). Anemia is also a predictor of adverse outcomes (21, 22). In our study, the results of the fitted curves suggested a negative linear relationship between hemoglobin levels and the incidence of diabetic foot ulcers.

The results of our subgroup analysis indicate an interaction between serum ferritin and DLEU. It is suggested that high levels of serum ferritin increased the incidence of DLEU risk. Previous studies have shown that ferritin significantly increased with increasing DFU severity (21, 23). Proinflammatory cytokines inhibit the absorption and mobilization of iron from storage into the circulation by down-regulating iron expression in intestinal epithelial cells, macrophages, and hepatocytes. This interference with iron metabolism leads to elevated ferritin expression, which shortens erythrocyte lifespan and impairs EPO production and function, ultimately inhibiting the proliferation and differentiation of normal erythroid progenitor cells (24).

There was significant difference between patients with and without anemia in terms of diabetic microvascular complications (neuropathy, retinopathy, nephropathy) and the related conditions (25–27). However, the mechanism linking anemia and DFU remains unclear. Possible mechanisms include the following: 1) Anemia reduces limb perfusion and exacerbates limb ischemia, which impairs tissue oxygenation and blood flow, ultimately delaying ulcer wound healing (28). 2) Additionally, the presence of anemia induces oxidative stress and hypoxemia with resultant delays in wound healing (29). 3) In DFU patients, the deformability of red blood cells is significantly reduced, and the proportion of non-deformable red blood cells is significantly increased, which can impede capillary flow and lead to thrombosis, which may result in delayed ulcer healing (30). 4) In patients with anemia, blood viscosity decreases, which impairs peripheral circulation, vascular smooth muscle response and EPO levels are destroyed, resulting in damage to the compensatory response of neovascularization and hindering wound healing (31). 5) Pro-inflammatory cytokines released in anemic patients affect iron metabolism, impair the production and function of EPO, and inhibit the proliferation and differentiation of normal red blood cell precursor (24). 6) Reduced tissue oxygenation can lead to increased production of free radicals, endothelial dysfunction and nerve damage (32). 7) Additionally, anemia can accelerate the progression of microvascular and macrovascular complications (28).

This clinical study examines the relationship between anemia and diabetic lower extremity ulcers (DLEU) in adult outpatients in the United States. The study found that Hb levels were a protective factor for DLEU. Anemia is a risk factor for DLEU.

However, the study has several limitations. Firstly, missing data were unavoidable due to the retrospective nature of the study and the data being extracted from the patients’ medical records. Secondly, it does not provide information on the potential causal effect of hemoglobin. Thirdly, larger and prospective studies are needed to overcome this limitation. The study has several limitations. Fourthly, the study was unable to determine other variables such as the severity of DFU and the cause of anemia. Finally, caution should be exercised when extrapolating these findings to other populations as the study focused on a specific population. Interventional studies are necessary to investigate whether clinical correction of anemia reduces the incidence of DLEU and improves its prognosis and prediction.

These findings may have clinical implications, such as better control of hemoglobin concentrations in diabetic patients, especially those diabetic lower extremity ulcers with anemia. It is also important to determine whether correcting anemia reduces the incidence of DLEU and to establish the optimal Hb level required to reduce the risk of diabetic lower extremity ulcers. Well-designed prospective studies are necessary to test the associations and confirm the relationship between anemia and the causation of diabetic lower extremity ulcers.

## Conclusion

The study found that hemoglobin level was a protective factor for DLEU, while anemia was an independent risk factor for DLEU in patients with diabetic lower extremity ulcers. Early identification of diabetic lower extremity ulcers risk provides an opportunity to delay or prevent disease onset. Prospective and multicenter studies are needed to explore whether anemia plays a direct role in the development, progression, or adverse outcomes of diabetic lower extremity ulcers.

Therefore, maintaining a higher concentration of hemoglobin is a protective factor that can prevent and ameliorate the development of DLEU.

## Data Availability

The original contributions presented in the study are included in the article/**Supplementary Material**. Further inquiries can be directed to the corresponding author.
