# Supplementary material for: Association between anemia and diabetic lower extremity ulcers among US outpatients in the National Health and Nutrition Examination Survey: a retrospective cross-sectional study
Source: Front Endocrinol (Lausanne). 2024 Aug 29;15:1387218. doi: 10.3389/fendo.2024.1387218 (PMC11390366; doi:10.3389/fendo.2024.1387218)
Supplement: Supplementary file 1 [file DataSheet1.docx]

Supplementary Material

# Supplementary Data

# Supplementary Figures and Tables

## Supplementary Figures

**Supplementary Figure 1.** Restrictive cubic spline of hemoglobin levels and diabetic foot ulcers (DFU).


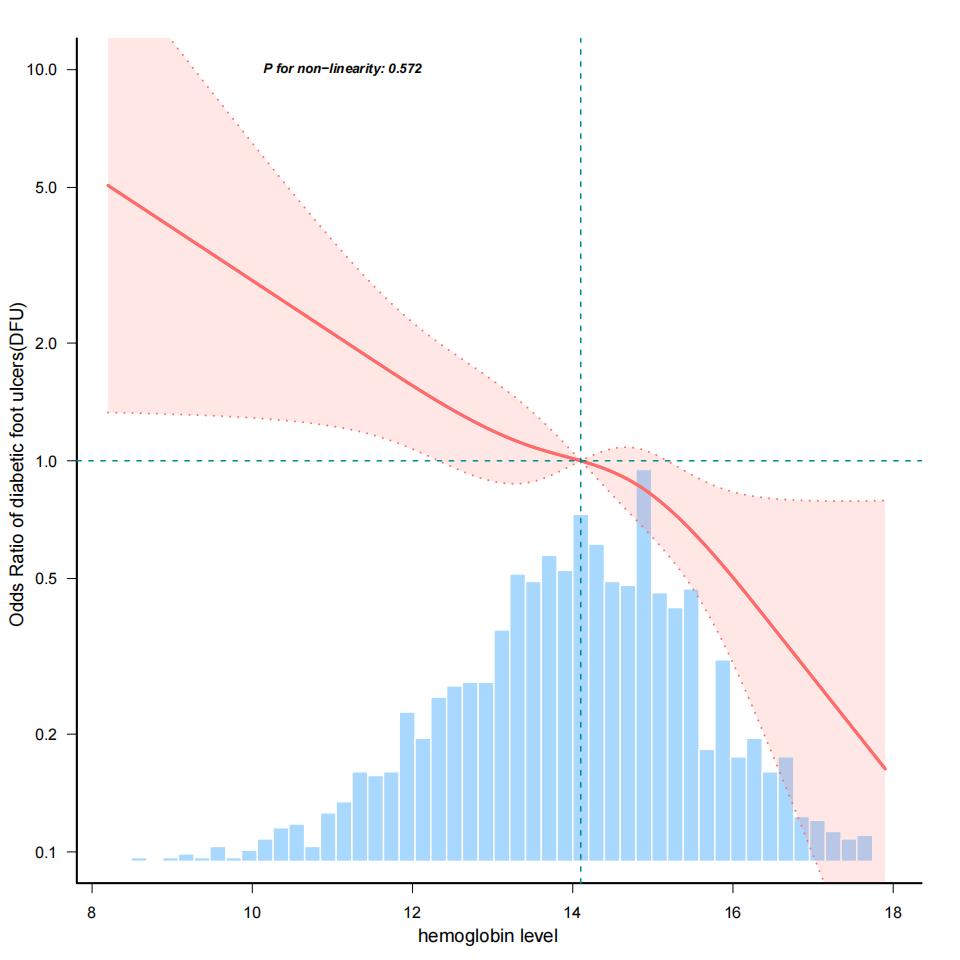


After conducting multivariate logistic regression analysis and smooth curve fitting, it was found that there is a negative association between hemoglobin levels and DFU incidence when all potential confounders were taken into account (non-linearity: p=0.572).
